# Repair-related molecular changes during recovery phase of ischemic stroke in female rats

**DOI:** 10.1186/s12868-022-00696-x

**Published:** 2022-04-12

**Authors:** Maryam Mostajeran, Lars Edvinsson, Hilda Ahnstedt, Kajsa Arkelius, Saema Ansar

**Affiliations:** 1 Division of Experimental Vascular Research, Department of Clinical Sciences, Faculty of Medicine, Lund University, Lund, Sweden; 2 Department of Neurology, McGovern Medical School at University of Texas Health Science Center at Houston, Houston, TX USA; 3 Applied Neurovascular Research, Neurosurgery, Department of Clinical Sciences, Faculty of Medicine, Lund University, Lund, Sweden

**Keywords:** Ischemic stroke, Molecular changes, Spontaneous recovery, Female, Repair mechanisms

## Abstract

**Background:**

Some degree of spontaneous recovery is usually observed after stroke. Experimental studies have provided information about molecular mechanisms underlying this recovery. However, the majority of pre-clinical stroke studies are performed in male rodents, and females are not well studied. This is a clear discrepancy when considering the clinical situation. Thus, it is important to include females in the evaluation of recovery mechanisms for future therapeutic strategies. This study aimed to evaluate spontaneous recovery and molecular mechanisms involved in the recovery phase two weeks after stroke in female rats.

**Methods:**

Transient middle cerebral artery occlusion was induced in female Wistar rats using a filament model. Neurological functions were assessed up to day 14 after stroke. Protein expression of interleukin 10 (IL-10), transforming growth factor (TGF)-β, neuronal specific nuclei protein (NeuN), nestin, tyrosine-protein kinase receptor Tie-2, extracellular signal-regulated kinase (ERK) 1/2, and Akt were evaluated in the peri-infarct and ischemic core compared to contralateral side of the brain at day 14 by western blot. Expression of TGF-β in middle cerebral arteries was evaluated by immunohistochemistry.

**Results:**

Spontaneous recovery after stroke was observed from day 2 to day 14 and was accompanied by a significantly higher expression of nestin, p-Akt, p-ERK1/2 and TGF-β in ischemic regions compared to contralateral side at day 14. In addition, a significantly higher expression of TGF-β was observed in occluded versus non-occluded middle cerebral arteries. The expression of Tie-2 and IL-10 did not differ between the ischemic and contralateral sides.

**Conclusion:**

Spontaneous recovery after ischemic stroke in female rats was coincided by a difference observed in the expression of molecular markers. The alteration of these markers might be of importance to address future therapeutic strategies.

**Supplementary Information:**

The online version contains supplementary material available at 10.1186/s12868-022-00696-x.

## Background

Stroke is a devastating disease and the leading cause of disability worldwide [1–3]. It is well documented that sex differences play a role in stroke incidence, outcome, and response to potential treatments [4]. For instance, functional recovery after stroke is worse in women, leaving women as the major burden of stroke-related disability and institutionalization [5]. Moreover, recombinant tissue plasminogen activator (rt-PA) treatment demonstrates a more favorable outcome in women and consequently less risk of intracerebral hemorrhage than men [6, 7].

Following the acute stroke injury, there is a spontaneous recovery process over time. Although this spontaneous functional improvement is limited, experimental stroke models have reported that cellular and molecular mechanisms underlying the endogenous brain repair mechanisms involve a set of highly interactive processes such as angiogenesis, scar formation and inflammation [8–10]. Each process consists of definite biomarkers, for instance, involvement of nestin in scar formation or recognition of tyrosine-protein kinase receptor Tie-2 in angiogenesis. Moreover, inflammatory markers [e.g., Interleukin (IL)-10] and growth factors like transforming growth factor (TGF) -β contribute as other repair-related molecular changes after stroke [8, 11, 12]. Signalling pathways are critical modulators of a variety of physiological and pathological processes including stroke recovery. Extracellular signal-regulated kinase (ERK) 1/2 and protein kinase B (Akt) pathways are contributing to the recovery phase associated with angiogenesis and protective effects of growth factors [13, 14]. Promoting these interactive molecular processes has been a relevant target in pre-clinical studies to aid stroke recovery. However, the majority of pre-clinical studies are performed in male rodents, a clear discrepancy when considering the patient situation in the clinic [15, 16].

To develop therapeutic strategies, it is significant to understand the pathophysiology during the recovery phase in females. Therefore, the aim of this study was to investigate the spontaneous functional recovery and repair-related molecular changes involved in the recovery phase two weeks after ischemic stroke in female rats. Transient middle cerebral artery occlusion (tMCAO) by intraluminal filament technique was used to induce experimental stroke. This model resembles a clinical situation where approximately 70% of human ischemic strokes affect parts of the brain nourished by the middle cerebral artery [17–19]. In addition, the intraluminal filament technique for inducing tMCAO is the most common method used in rodents for experimental ischemic stroke [18, 20].

## Results

Female Wistar rats (12-week-old) were monitored for two to three consecutive estrous cycles by collecting vaginal smears and examining the types of cells present. Animals that were under low influence of 17β-estradiol underwent tMCAO (120 min) followed by reperfusion 14 days post tMCAO. Physiological parameters including mean arterial blood pressure (119 ± 11.1 mm Hg), pO_2_ (15.6 ± 2.4 kPa), pCO_2_ (7.3 ± 0.05 kPa), pH (7.3 ± 0.05), blood glucose (13.2 ± 1.5 mmol/L) and body temperature (36.6 ± 0.6 °C) were within acceptable limits during the surgical procedure. Humane endpoints were followed according to the study plan (e.g., monitoring body weight) to minimize pain and suffering and none of the animals were excluded from the study based on humane endpoint evaluation. Two animals died outside planned euthanasia or humane endpoints and post-surgical procedures while eight animals went through the entire planned experimental time course.

## Spontaneous functional recovery within 14 days after tMCAO

**Fig. 1:**
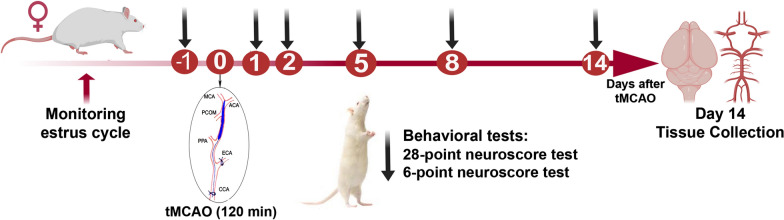
Experimental design. Transient middle cerebral artery occlusion (tMCAO) was induced in female Wistar rats at day 0 and the in vivo part of the study was designed for a period of two weeks. tMCAO was induced in animals that were under low influence of 17β-estradiol during their estrous cycle. Animals were acclimatized with animal facility conditions for 5 days before any in vivo experiments. At day 14 after reperfusion, the animals were euthanized and decapitated. *CCA* common carotid artery; *ECA* external carotid artery; *ICA* internal carotid artery; *MCA* middle cerebral artery

Neurological deficit was evaluated according to the following established composite tests in stroke models: 28-point [21] and 6-point [22, 23] neuroscore tests at day 1 (pre-stroke), and at day 1, 2, 5, 8 and 14 post tMCAO and during reperfusion. A gradually and significant recovery up to day 14 was observed by both tests (Fig. 1A, B). In 28-point test, the most neurological deficits were observed at day 2 post stroke. These deficits were gradually and significantly recovered up to day 14 [animals’ neuroscore for day 2: 15 (12.25–20) vs 21.5 (17.5–24) for day 14, p < 0.01]. Evaluation of the 6-point test demonstrated that female rats elicit the greatest neurological damage on day 1 post stroke. Significant recovery from neurological deficits based on a6-point test was seen at day 14 compared to day 1 post stroke (2.5 (2–3) vs 3.5 (3–4) respectively, p < 0.05). However, neurological deficits still significantly differed at day 14 compared to the day before stroke (21.5 (17.5–24) for 28-point score test and 2.5 (2–3) for 6-point score test, p < 0.05). To verify brain damage, silver infarct staining was performed on brain Sects. 14 days after tMCAO. The extent of brain damage on the ipsilateral side was 17.51 ± 7.33% (n = 8) which corresponded to and infarct volume of 118.12 ± 60.67 mm^3^. Infarct distribution is shown in representative images of silver infarct staining (Fig. 1C).

## ERK1/2 and Akt pathways were affected at day 14 after tMCAO

**Fig. 2:**
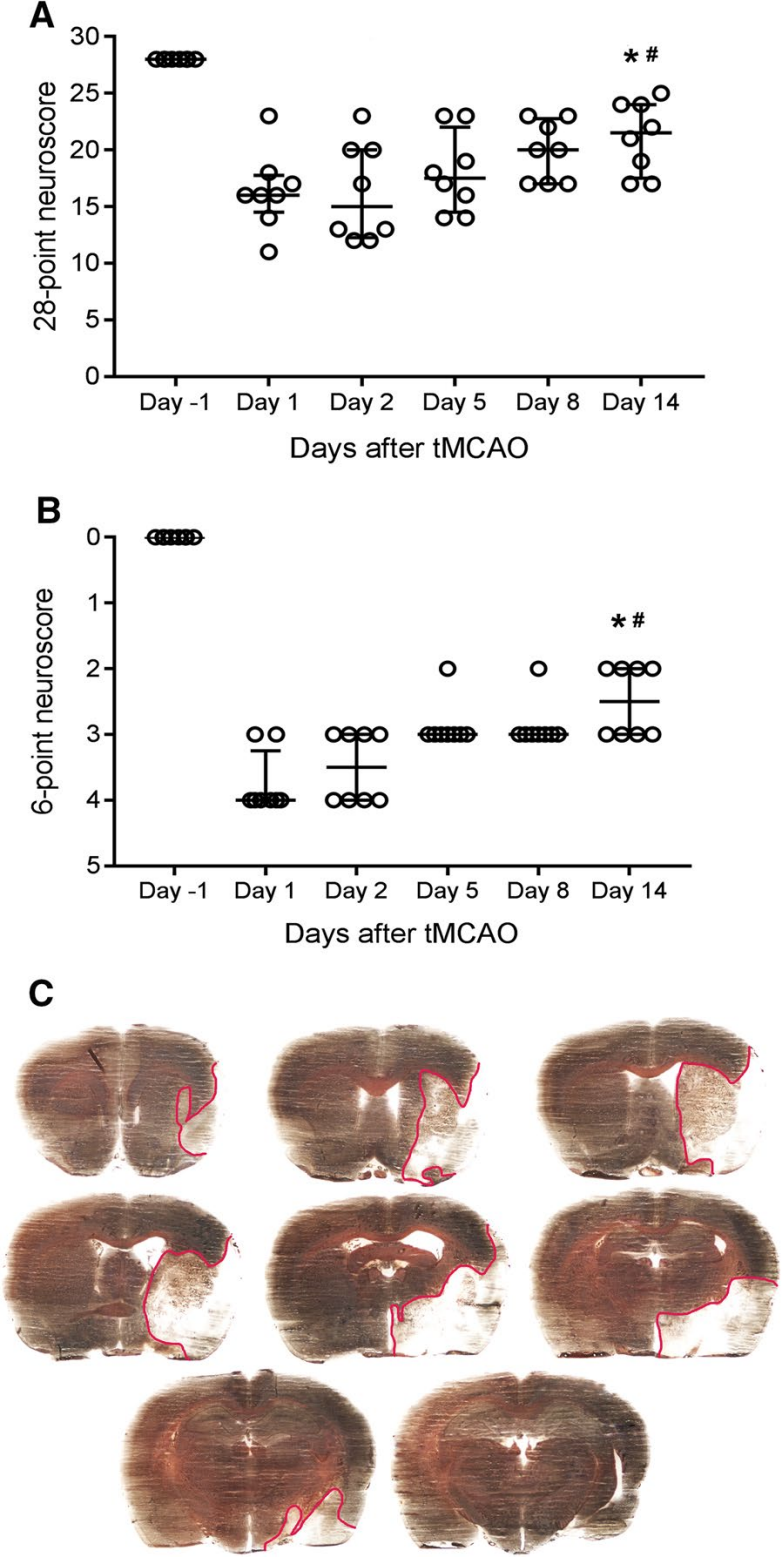
Spontaneous functional recovery after ischemic stroke. **A** Spontaneous recovery from day 2 to 14 after tMCAO was observed by 28-point test **B** neurological score of animals from 0 for healthy function to 5 for death overnight is shown. Neurological function of the animals was significantly affected by tMCAO, however, the animals showed significant functional improvement at day 14 after tMCAO. *Significance level at p < 0.05 for comparison between day -1 (pre-stroke) and day 14. ^#^Significance level at p < 0.05 for comparison between day 1 (for 6-point test) and 2 (for 28-point test) vs day 14. Number of rats in both tests; n = 8. **C** Infarct distribution in representative silver infarct staining of brain sections. Lesioned area is marked in red on right hemisphere

The ERK1/2 and Akt pathways were evaluated at day 14 after experimental stroke in female rat brains. The results demonstrate that the phosphorylation (p) state of ERK1/2 relative to total (t) ERK1/2 normalized to β-actin was significantly increased in the peri-infarct and ischemic core compared to contralateral side at day 14 after tMCAO (Fig. 2A, B). The expression levels revealed significant decrease in ratio of t-ERK1/2 to β-actin in ischemic core compared to control side. However, this ratio remained unchanged in the peri-infarct region vs control side. The p-Akt/t-Akt relative to β-actin was significantly increased at day 14 after tMCAO in peri-infarct and ischemic core regions compared to contralateral side (p < 0.05). However, the level of t-Akt significantly decreased after ischemic stroke in both ipsilateral peri-infarct and ischemic core compared to contralateral side at day 14 after tMCAO as shown in Fig. 2C, D.

## Expression of markers involved in angiogenesis, scar formation and inflammation

**Fig. 3:**
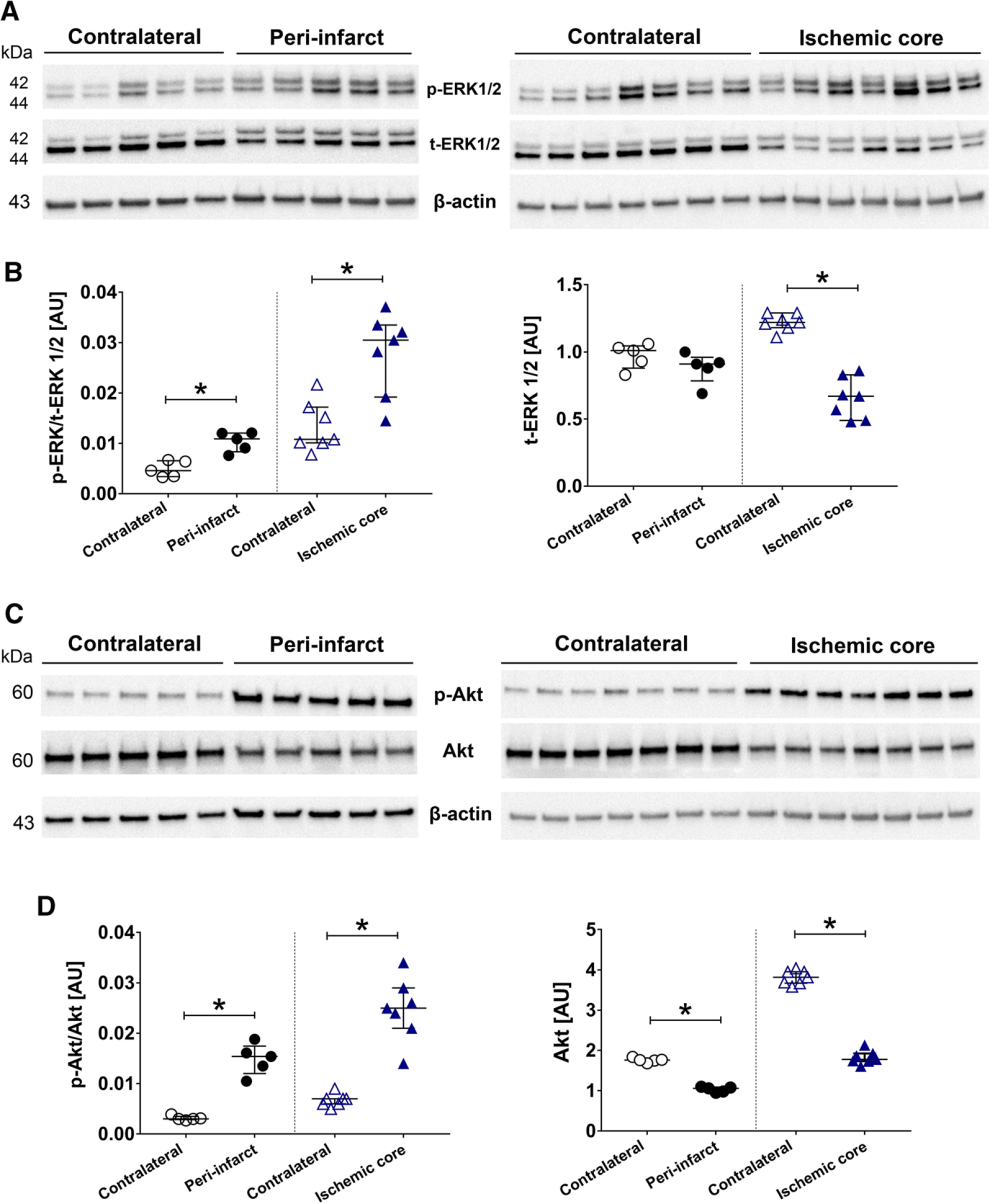
ERK1/2 and Akt pathways are affected at day 14 after tMCAO. **A** Representative western blots of p-ERK1/2 and t-ERK1/2 for peri-infarct and ischemic core vs. contralateral samples. **B** Quantification of p-ERK1/2 (normalized to t-ERK1/2 and β-actin—left graph) and t-ERK1/2 (normalized to β-actin—right graph) in peri-infarct and ischemic core compared to contralateral. **C** Representative western blots of p-Akt and Akt for peri-infarct vs contralateral (left panel) and ischemic core vs contralateral (right panel). **D** Quantification of p-Akt (normalized to Akt and β-actin) in peri-infarct and ischemic core showed significant increase compared to their related contralateral samples (left graph). Akt expression was significantly lower in peri-infarct and ischemic core compared to contralateral (right graph). Number of animals for ischemic core; n = 7, number of animals for peri-infarct; n = 5. *AU* arbitrary units, *Significant differences (p < 0.05). The presented western blot images are cropped and the full-length blots/gels are presented in Additional file 2: Figure S1

The expression of nestin, NeuN, TGF-β, Tie-2 and IL-10 at day 14 after tMCAO were examined in the ischemic region and the contralateral side of brain tissue. There was a significant increase in expression of nestin in both peri-infarct and ischemic core compared to contralateral side. However, NeuN was significantly lower in both of the studied ischemic regions within the ipsilateral side compared to control side (Fig. 3A, B). Quantitative analysis of TGF-β bands showed enhanced expression in the ischemic core compared to the contralateral side (p < 0.05). Although expression of TGF-β increased in the peri-infarct region, this change was not significant when compared to control side. Expression of Tie-2 and IL-10 proteins were unchanged compared to contralateral side in peri-infarct and ischemic core region (Fig. 3C, D). β-actin was used as a loading control and data for each molecular target were normalized by β-actin for the statistical calculations and comparisons.

## TGF-β significantly increased in occluded MCAs during recovery phase

**Fig. 4:**
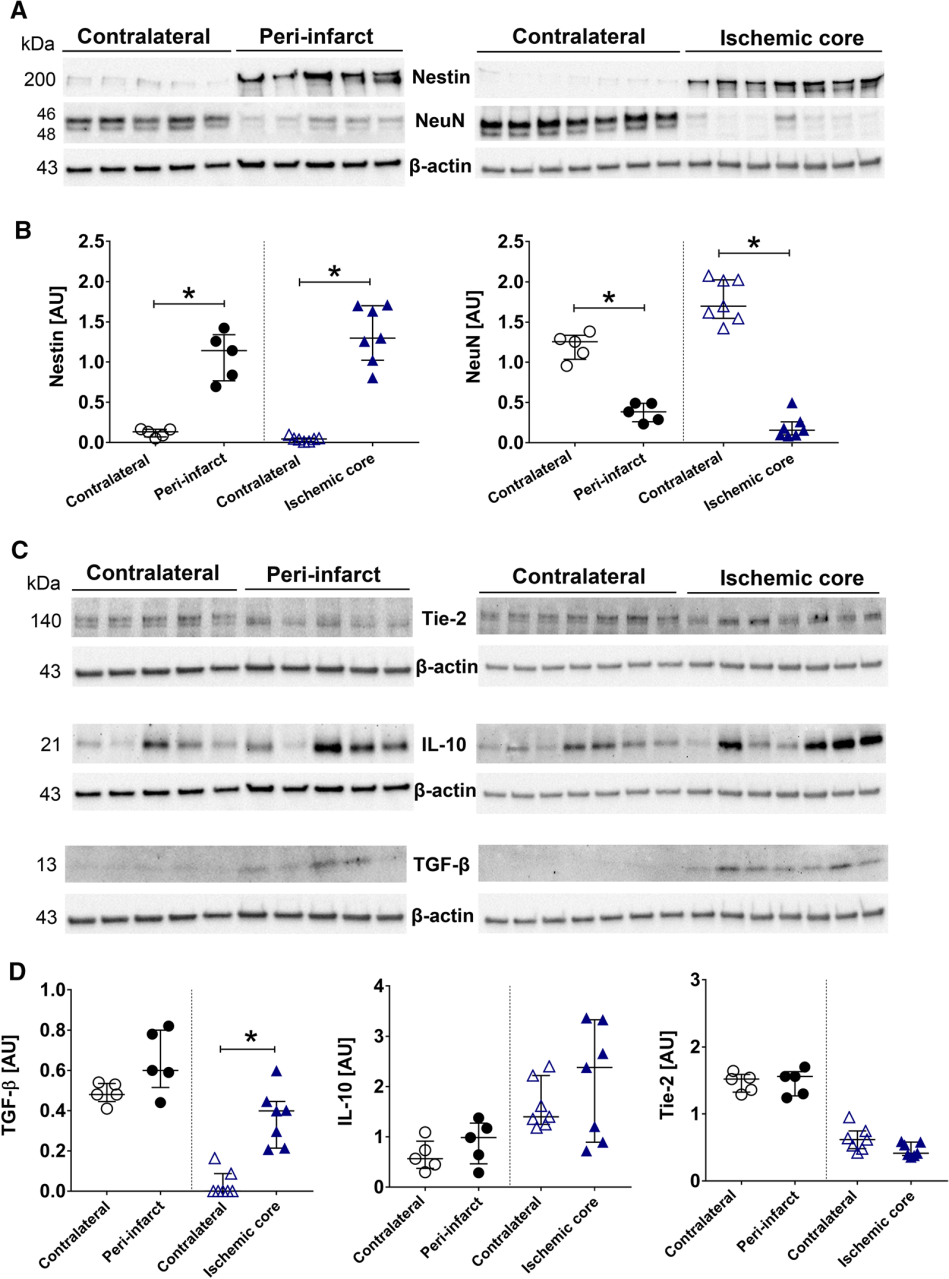
Expression of Nestin, NeuN, Tie-2, IL-10 and TGF-β in brain at day 14 after tMCAO. **A**, **B** Representative western blots and quantification of NeuN, nestin and TGF-β in peri-infarct and ischemic core vs. contralateral side. **C**, **D** Representative western blots and quantification of Tie-2 and IL-10 in peri-infarct and ischemic core vs contralateral side. All markers were normalized to β-actin. Ischemic core; n = 7, peri-infarct; n = 5. *AU* arbitrary units, *Significant differences between peri-infarct or ischemic core and contralateral (p < 0.05). The presented western blot images are cropped and the full-length blots/gels are presented in Additional file 3: Figure S2 (Nestin and NeuN) and Additional file 4: Figure S3 (Tie-2, IL-10 and TGF-β)

Expression of TGF-β was evaluated in left (non-occluded) and right (occluded) MCAs by immunohistochemistry at day 14 after tMCAO. Qualitative assessment of acquired images showed that TGF-β was expressed as a cytoplasmic marker both in the smooth muscle cell layer and in endothelial cells of MCA (Fig. 4A). Moreover, expression of TGF-β in the smooth muscle layer of occluded MCAs significantly increased compared to non-occluded side using mean fluorescence intensity measurement (Fig. 4B, p < 0.05).

## Discussion

In the present study, we showed that spontaneous functional recovery in female rats after ischemic stroke is accompanied by a significantly higher expression of nestin, p-Akt, p-ERK1/2 and TGF-β in ischemic regions compared to contralateral side at day 14. Spontaneous functional recovery is usually seen in weeks to months after a stroke in human patients [8]. Studies in animal models of stroke have provided insights into this spontaneous functional recovery and the sensorimotor sequela following rat models of cerebral ischemia in male rats have been studied previously [21]. However, studies in female animals are limited and, hence it is important to include them in preclinical stroke research [24].

In this study, a significant spontaneous recovery was demonstrated during a period of 2 weeks after tMCAO in female rats. However, we did not observe a plateau level in the neurological functions of female rats at day 14. These findings are in agreement with previous studies in male animals [25, 26] and clearly indicates that an active recovery process is still ongoing at day 14. To shed more light on the point where functional recovery becomes more stable, the time-window for the study should be expanded for at least 1 month in rats since studies suggest that these later time points is probably more representative for the clinic. Additional behavioural tests should be performed to get a more comprehensive view of the recovery process. The tests used in this study assess a variety of responses such as motor, sensory, reflex and balance.

Animal studies also provide further details of the aligned cellular and molecular mechanisms with spontaneous recovery after stroke including angiogenesis, scar formation, inflammation and signalling pathways. ERK1/2 and Akt are two important pathways contributing to the pathophysiology of stroke. We showed in our previous studies that ERK1/2 is activated during acute phase of experimental stroke and involves destructive mechanisms i.e. inflammation and upregulation of vasoconstriction receptors [27, 28]. By early inhibition of ERK1/2 pathway after tMCAO, infarct size was reduced and neurological functions were improved both during acute and subacute phase [25, 26, 29]. We have also noticed that ERK1/2 is activated following two weeks after tMCAO in male rats when recovery processes were ongoing. Interestingly, early inhibition of the pathway did not affect its later stage activation but also benefited recovery mechanisms [26]. An increase of p-ERK1/2 in the peri-infarct region which indicates activation of this pathway was also observed in the present study 2 weeks post-stroke in female rats. Here, we have revealed a notable activation of p-ERK1/2 in parallel with active functional recovery. The involvement of ERK1/2 pathway in the pathophysiology and recovery of ischemic stroke was also reviewed elsewhere [13, 30]. We suggest that p-ERK1/2 is an important modulator of stroke pathophysiology; however, the timing in which this pathway is studied is very important as ERK1/2 can have destructive effects in the early-stage however it is involved in the molecular mechanisms underlying the event of recovery in a later stage for both female and male rodents.

Various markers including growth factors [31] or anti-inflammatory cytokines [32] exert their beneficial effects by activation of Akt pathway after experimental stroke [33]. Our results showed significant reduction of t-Akt protein but increase in the ratio of p-Akt (ser-473) to t-Akt in ischemic regions compared to control side. A previous study suggested that phosphorylation of Akt (ser-473) does not necessarily correlate with its kinase activity since Akt is phosphorylated by neuronal insults and thereafter an endogenous inhibitor can extinguish its activity [34]. Phosphorylation of Akt Ser-473 promotes a Lys-48-linked polyubiquitination of Akt which leads to its rapid degradation [35]. Therefore, manipulation of Akt pathway for stroke therapy should be carefully addressed. We hypothesize that in absence of survival factors, Akt can undergo degradation via phosphorylation of Ser-473 at longer time point after stroke in female rats; however, survival factors like growth factor can switch this phosphorylation towards Akt stability and activation. Evaluation of downstream targets of Akt can provide more insights about contribution of Akt in recovery phase after stroke.

TGF-β has anti-inflammatory [36] and anti-apoptotic actions, promotes scar formation and angiogenesis [37–40] through Akt and ERK1/2 pathways [13, 14]. However, most of the mechanistic studies evaluated male animals and studies addressing females are few. In this study, TGF-β levels were significantly higher in ischemic area compared to control side in female at day 14. Our observation is supported by a previous study showing that astrocytes control neuroinflammation via TGF-β signalling and preserve brain function 2–3 days after stroke in female mice [41]. Moreover, TGF-β signalling in the brain after stroke is reported to be equivalent in males and females peaking on day 7 in mice [42].

We report for the first time and to our knowledge, a significant increase in the expression of TGF-β in the smooth muscle layer of the MCAs subjected to stroke compared to non-occluded MCAs at day 14 post-stroke in female rats. The cerebral vasculature crucially participates in brain recovery processes [43] and evidence has shown that angiogenesis supports restored perfusion in the ischemic border after cortical stroke in female rats [44]. The expression of TGF-β both in brain and MCAs together with the activation of ERK1/2 pathway in female rats can suggest that TGF-β implements its roles through this pathway either in angiogenesis or neuroprotection similar to males. However, the direct interaction of these components in functional improvement after stroke in female rats has not been addressed in this study.

Moreover, angiogenesis involves the interaction of other components including Tie-2, angiopoietin (Ang)-1, 2 and vascular endothelial growth factor [39]. We observed no difference for Tie-2 in ischemic hemisphere compared to contralateral in tMCAO female rats. Similar results have been observed in male rats for the same studied time-points following MCAO [38]. However, induction of Tie-2 and Ang proteins were shown in other stroke studies [40, 45]. Regardless of the controversy in data obtained in male experimental stroke models, we speculate that occurrence of angiogenesis upon ischemia is accompanied by changes in the endogenous expression of other angiogenic markers but not Tie-2 in female rats.

We observed that the expression of nestin is significantly increased in the ischemic regions compared to the control side during recovery phase. Nestin, an intermediate filament protein [46], is a marker for new neurons and has also been expressed in astrocytes surrounding infarct with formation of gliotic scar, preventing infarct to expand and contributing to recovery of neurological functions [47, 48]. It was demonstrated that formation of astrocytic scar aids axonal regeneration in central nervous system [49]. Our data is in parallel with male studies suggesting the expression of nestin may contribute to formation of gliotic scar after stroke. Enhanced adult neurogenesis is a prominent delayed effect and important contribution to functional recovery after stroke [50]. However, the adult neurogenesis is a complex phenomenon and not fully understood. Doublecortin (DCX) positive cells are good markers to be used for adult neurogenesis since these cells are the precursors of mature neurons. It has been demonstrated that ablation of DCX neuroblasts worsened functional recovery [51]. In future studies, an evaluation of the recruitment of neuroblast from subventricular zone to striatum should be included to obtain a more comprehensive picture of the neurogenesis processes.

Clinical and preclinical studies (mostly performed in male animals) report opposite results on the effect of IL-10 in response to stroke [52]. We did not observe any difference in IL-10 expression between ischemic and non-ischemic sides in female rat brain during recovery phase of stroke. One reason for this observation could be sample size and the considerable variation of IL-10 seen between individuals. Other explanation could be that evaluation of IL-10 was performed at one time-point during recovery phase. Previous study showed smaller infarct volume in female vs male mice at day 4 after tMCAO was accompanied by an increased distinct population of IL-10-secreting CD8^+^CD122^+^ T-suppressor cells in female mice as an underlying mechanism [53]. However, another study showed that higher level of IL-10 was associated with poorer outcome in female patients but not in males [11]. The controversy in the role of IL-10 in ischemic stroke and fewer studies in females illustrate the necessities of more targeted studies to evaluate precise contribution of IL-10 following acute brain injuries [52].

This is one of the first long-term studies performed on female rats after stroke investigating the recovery process, however to better understand the underlying mechanisms during the recovery phase of stroke, more markers should be considered in future studies representing the important processes such as neurogenesis, angiogenesis and synaptogenesis. Microglial activation has a significant important role in these processes and coincides with brain plasticity after stroke. To achieve a holistic assessment and understand the mechanisms involved in recovery markers for microglia and astrocytes should be evaluated. In our study we showed an enhanced expression of TGF-β that are known to be produced by microglia cells. To achieve a more extensive evaluation and a broader perspective of the functional recovery processes additional neurological tests and an extended time-window of the study up to at least 4 weeks should be performed.

## Conclusions

Our study highlights the importance of studying females in stroke research and it provides evidence for involvement of important processes during recovery phase. We clearly demonstrate that a spontaneous functional recovery is coincided by significantly higher expression of nestin, p-Akt, TGF-β and activation of ERK1/2 in ischemic regions following ischemic stroke in female rats. These findings might be part of the underlying mechanisms during the recovery phase of stroke and can be addressed to promote brain remodelling, aid stroke recovery and develop new therapeutic strategies in females. Spontaneous recovery will not be enough and there is an urgent need to develop new interventions that enhance functional recovery and counter neurological deficits in stroke survivors.

## Materials and methods

### Animal preparation and study design

**Fig. 5:**
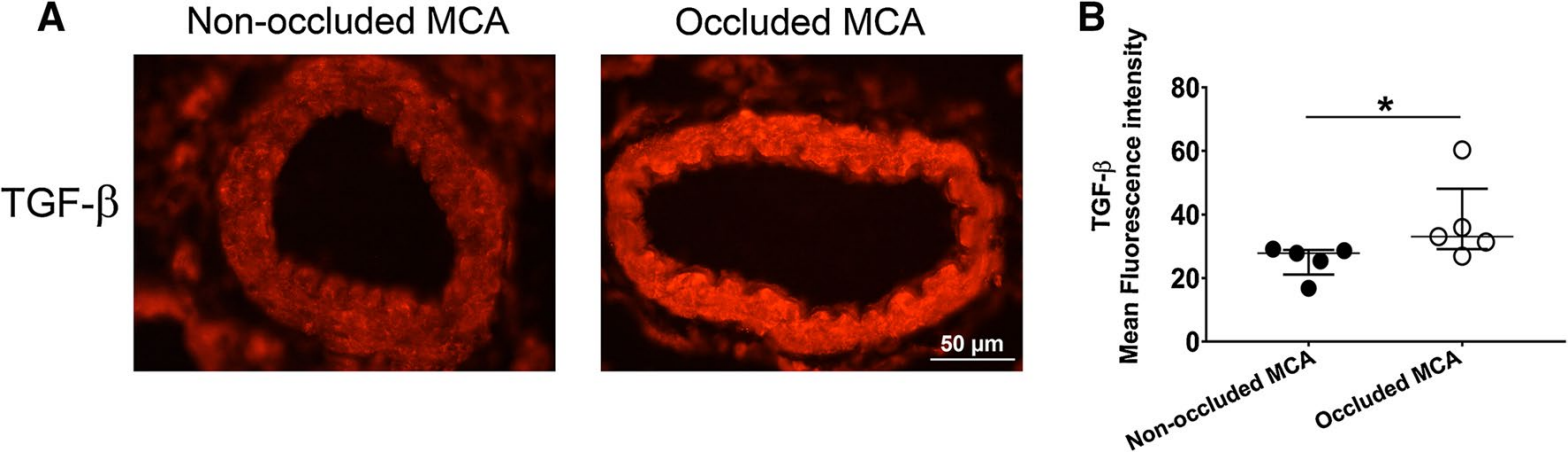
Ischemia significantly increased expression of TGF-β in the smooth muscle cell layer of occluded MCAs. **A** Representative image of TGF-β expression at day 14 after tMCAO in the occluded and non-occluded MCAs. **B** Quantification of TGF-β mean fluorescence intensity in occluded vs non-occluded MCAs at day 14 after tMCAO; n = 5, *p < 0.05

Twelve-week-old female Wistar rats were purchased from Charles River laboratories (Sulzfeld, Germany), maintained in animal conventional facility (temperature: 21 °C and humidity: 55%) with 12 h light/dark cycle (light cycle: 7 a.m.–7 p.m.) and housed in cage type III H, two in each cage, with free access to food (Special Diets Services RM1) and water. Sizzelnest was used as bedding material and the cages were enriched with chew stick and paper roll. After minimum 5 days of acclimatization in animal conventional facility, animals were monitored for two–three consecutive estrous cycles by determination of the cell types present in vaginal smear [54, 55]. Animals that were under low influence of 17β-estradiol were subjected to the intraluminal filament technique [26, 29] to induce transient middle cerebral artery occlusion. This is to minimize hormonal fluctuation effects at the time of surgery as estradiol has been shown to be neuroprotective in ischemic stroke [4]. In total ten animals were utilized in the study, two of the animals died outside planned euthanasia or humane endpoints and post-surgical procedures while eight animals went through the entire planned experimental time course. The experimental design of the study is shown in Fig. 5.

### Characterization of the estrus cycle

The estrous cycle was monitored daily (between 9 and 9.30 a.m.) through vaginal smears collected by use of cotton swabs and spreading onto positively charged glass slides. Hematoxylin/eosin staining on the smears according to a standard protocol (4 min/30 s) made it possible to characterize the types of cells present (described in detail by [54]) and consequently to determine the day of the estrous cycle for each individual rat. In proestrus, the presence of large round nucleated epithelial cells, often in clusters, can be distinguished. Estrus is characterized by a large number of non-nucleated needle-like cornified cells. A combination of round epithelial cells, small leukocytes and cornified cells are observed in diestrus 1 and 2 [25, 55].

### Transient middle cerebral artery occlusion (tMCAO)

Induction of tMCAO was performed as previously described [56]. Briefly, the right external carotid artery was ligated permanently and a silicon rubber-coated monofilament (Doccol Corporation, MA, USA) was inserted through the right internal carotid artery and advanced to the bifurcation of the middle cerebral artery (MCA) causing a drop in cerebral blood flow (CBF). Average Laser-Doppler flow reduction of CBF was 74.12 ± 10.16% with lower inclusion limit of 60% reduction (AD instruments, Australia). The animals were evaluated by 6-point test [22, 23] after 2 hours of occlusion. Only animals receiving a score of 4 were re-anesthetized to proceed with the reperfusion phase. To achieve reperfusion, the filament was removed and the increase of CBF during reperfusion was simultaneously recorded and verified by Laser-Doppler flowmeter. All efforts were made to minimize suffering; the rats received Marcaine (1.25 mg/kg, AstraZeneca) at the site of incisions as analgesics. A subcutaneous injection of 10 ml of isotonic saline for rehydration was also given at the end of the surgery. Humane endpoints were also considered within the study plan; animals’ body weight was monitored within two weeks post operation as a general condition check-point. Posture, activity and social behaviour of the animals were also checked by the experimenter and staff at conventional animal facility. Animals that lost more than 15% of their pre-operational body weight together with showing inactivity, body cramping, not responding to stimulation and not being able to do righting reflex were excluded from the study. Day 14 post-reperfusion was chosen as the end point of the in vivo part of the study. All animal surgeries were performed between 8 a.m. and 6 p.m. in the laboratory equipped with ventilation and rodent anaesthetic system.

### Neurologic examination

Animals were kept for 14 days after tMCAO and during this time, evaluated by neurological examination at day 1, 2, 5, 8 and 14 after tMCAO according to established composite tests in stroke models: 28-point [21] and 6-point [22, 23] tests. The rating was performed by an experienced scorer starting from 0 for severe impairment to the maximum of 28 for healthy function of the animal in the 28-point test. In the 6-point test, the animal was graded from 0 for healthy function to 5 for death overnight.

### Tissue harvesting

The rats were euthanized by CO_2_ and decapitated at day 14 after tMCAO accordingly to our ethical permit. Brains were quickly removed and immersed in ice-cold bicarbonate buffer [57]. Right and left middle cerebral arteries (MCAs) were dissected out and fixed in 4% paraformaldehyde in phosphate-buffered saline (PBS, pH = 7.2) for 2 h at 4 °C followed by cryopreservation in 10% and 25% sucrose in PBS. Thereafter, they were embedded in TissueTEK (Gibco, Invitrogen A/S, Taastrup, Denmark). After dissecting out MCAs, brains were quickly frozen in -20 °C methyl butane. All the tissues were stored at − 80 °C until use for in vitro part of the study.

### Silver infarct staining

Frozen brains were cut (40-µm) at an interval of 400 µm on a cryostat (Microm HM 560; Thermo Scientific, MA, Waltham, USA). The first section of every ten sections was used for silver infarct staining and the other nine in the infarct region were used for western blot. The sections were stained according to an established method [58]. Each section was photographed (Infinity 2 microscope camera, Lumenera Corporation Ottawa, Ontario, Canada) and infarct size was reported as percentage of ipsilateral volume using ImageJ software (http://rsb.info.nih.gov/ij/).

### Western blot

Brains were cut into 40-µm cryosections and tissues were collected based on three different regions; ischemic core, peri-infarct and contralateral as described previously [26]. The protein samples obtained from brain tissues (15 µg protein/lane) were loaded on a 4–20% gradient TGX precast gel (Bio-Rad Laboratories, Hercules, CA, USA), transferred onto a nitrocellulose membrane and incubated with primary antibodies overnight at 4 °C. Subsequently, the membranes were incubated with appropriate secondary antibodies for 1 h at room temperature. Detailed description of antibodies is provided in Table 1. The protein bands of interest were visualized by enhanced chemiluminescent staining in a Fujifilm LAS-100 Luminescent Image Analyzer (Stamford, CT, USA). The membranes were re-probed with β-actin: peroxidase conjugated (1:50,000, A3854, Sigma-Aldrich, MO, USA) for loading control.

**Table 1:** Primary and secondary antibody information for western analysis

Antibodies	Dilution	Supplier (catalogue no.)
Primary antibodies		
Akt	1:1000	Cell signaling (9272S)
p-Akt (Ser473)	1:1000	Cell signaling (4058S)
p-ERK1/2	1:2000	Cell signaling (9101)
t-ERK1/2	1:4000	Cell signaling (9107)
IL-10	1:1000	Abcam (ab25073)
Nestin	1:1000	Abcam (ab6142)
NeuN	1:10,000	Abcam (ab104224)
TGF-β	1:250	Abcam (ab66043)
Tie-2	1:200	SantaCruz (sc-9026)
Secondary antibodies		
Anti-rabbit IgG:peroxidase	1:2000	Cell signaling (7074)
Anti-mouse IgG:peroxidase	1:2000	Cell signaling (7076)

### Immunohistochemistry

Embedded and frozen MCAs were cut on a cryostat (Microm HM 560; Thermo Scientific, MA, Waltham, USA) into 10-µm sections. Thereafter, the sections were permeabilized in PBS containing 0.25% Triton X-100, blocked and incubated overnight at 4 °C with rabbit anti-TGF-β (1:250, Abcam, ab66043, Cambridge, UK). Then, the secondary antibody was applied (1 h, room temperature, dark); Cy^3^ donkey anti-rabbit (1:200, Jackson ImmunoResearch, 711–165-152, West Grove, PA). Both antibodies were diluted in PBST + 1% bovine serum albumin. Sections were finally mounted with Vectashield mounting medium (Vector Laboratories Inc., Burlingame, CA, USA) containing 4′,6-diamidino-2-phenylindole (DAPI) that satins nuclei. Negative controls were performed by omitting primary antibody. The experiment was repeated to ensure reproducibility. Immunoreactivity was visualized at the appropriate wavelength with an epifluorescence microscope (Nikon 80i; Tokyo, Japan) and photographed with an attached Nikon DS-2MV camera with 40 × lenses.

### Statistics

Data are expressed as median with interquartile range (IQR) except for body weight and physiological parameters where mean ± standard deviation (SD) is reported. SPSS (IBM Corp. Released 2017, Version 25. Armonk, NY, USA) was used to perform statistical analyses, n refers to number of rats and p < 0.05 was considered as significant.

#### Composite tests

Spontaneous functional recovery from neurological deficits were analyzed by comparing the score of animals at day-1 (pre-stroke), day 2 and day 14 after stroke for the 28-point neuroscore test and day-1 (pre-stroke), day 1 and day 14 after stroke for the 6-point test by using Friedman test and Wilcoxon post-hoc with Bonferroni correction (n = 8 for each test).

#### Western blot

Contralateral samples together with their respective peri-infarct (n = 5) or ischemic core samples (n = 7) were run on same blots. Two-tailed Wilcoxon Signed Ranks test were performed to analyze the difference in the expression of markers between contralateral and peri-infarct or between contralateral and ischemic core as related samples.

#### Immunohistochemistry

Staining was evaluated with an expert blinded to the study to localize positive immunoreactivity. Moreover, fluorescence intensity was measured in the smooth muscle layer using ImageJ. The difference in mean intensity between right (occluded) and left (non-occluded) MCAs of each individual was analyzed by two-tailed Wilcoxon Signed Ranks test as related samples (n = 5).

## Supplementary Information

**Additional file 1: Checklist S1.** The ARRIVE guidelines 2.0: author checklist.

**Additional file 2: Figure S1.** Raw images for Western blot membranes corresponding to Fig. 3A, B in the manuscript. 2A: phosphorylated ERK1/2, total ERK1/2 and β-actin corresponding to Fig. 3A in the manuscript. Fig. 3C: phosphorylated Akt, total Akt, and β-actin corresponding to Fig. 3A in the manuscript. In all peri-infarct vs. contralateral samples (left columns) the lanes labelled with number 1–5 are contralateral samples and lanes labelled number 6–10 are their respective peri-infarct samples. The lanes marked with X were test samples used for the accuracy of the experiment and were not included for statistical analysis; therefore, they were omitted in final figures. In all contralateral versus ischemic core samples (right columns), the lanes labelled number 1–7 are contralateral samples and the lanes labelled number 8–14 are their respective ischemic core samples. Arrows show the band for the protein of interest according to molecular weight. The other bands are nonspecific which could be due to the many different cell types in brain tissue. Molecular weight marker was loaded in all experiments. *M* molecular marker.

**Additional file 3: Figure S2.** Raw images for Western blot membranes corresponding to Fig. 4A in the manuscript. 3A: Nestin, NeuN and β-actin corresponding to Fig. 4C in the manuscript. In all contralateral samples versus peri-infarct (left columns) the lanes labelled with number 1–5 are contralateral samples and lanes labelled number 6–10 are their respective peri-infarct samples. The lanes marked with X were test samples used for the accuracy of the experiment and were not included for statistical analysis; therefore, they were omitted in final figures. In all contralateral versus ischemic core samples (right columns), the lanes labelled number 1–7 are contralateral samples and the lanes labelled number 8–14 are their respective ischemic core samples. Arrows show the band for the protein of interest according to molecular weight. The other bands are nonspecific which could be due to the many different cell types in brain tissue. Molecular weight marker was loaded in all experiments. *M* molecular marker.

**Additional file 4: Figure S3.** Raw images for Western blot membranes corresponding to Fig. 4C in the manuscript. 3C: Tie-2, IL-10, TGF- β and β-actin corresponding to Fig. 4C in the manuscript. In all contralateral samples versus peri-infarct (left columns) the lanes labelled with number 1–5 are contralateral samples and lanes labelled number 6–10 are their respective peri-infarct samples. The lanes marked with X were test samples used for the accuracy of the experiment and were not included for statistical analysis; therefore, they were omitted in final figures. In all contralateral versus ischemic core samples (right columns), the lanes labelled number 1–7 are contralateral samples and the lanes labelled number 8–14 are their respective ischemic core samples. Arrows show the band for the protein of interest according to molecular weight. The other bands are nonspecific which could be due to the many different cell types in brain tissue. Molecular weight marker was loaded in all experiments. *M* molecular marker.

## Data Availability

The data that support the findings of this study are available upon reasonable request.

## Abbreviations

Ang: Angiopoietin
CBF: Cerebral blood flow
ERK1/2: Extracellular signal-regulated kinase 1/2
IQR: Interquartile range
IL-10: Interleukin 10
MCA: Middle cerebral arteries
NeuN: Neuronal specific nuclei protein
PBS: Phosphate-buffered saline
Akt: Protein kinase B
SD: Standard deviation
t-PA: Tissue plasminogen activator
TGF-β: Transforming growth factor
tMCAO: Transient middle cerebral artery occlusion
Tie-2: Tyrosine-protein kinase receptor Tie-2
