# Supplementary material for: Repair-related molecular changes during recovery phase of ischemic stroke in female rats
Source: BMC Neurosci. 2022 Apr 12;23:23. doi: 10.1186/s12868-022-00696-x (PMC9004052; doi:10.1186/s12868-022-00696-x)
Supplement: Supplementary file 2 — Additional file 2: Figure S1. Raw images for Western blot membranes corresponding to Fig. 3A, B in the manuscript. 2A: phosphorylated ERK1/2, total ERK1/2 and β-actin corresponding to Fig. 3A in the manuscript. Fig. 3C: phosphorylated Akt, total Akt, and β-actin corresponding to Fig. 3A in the manuscript. In all peri-infarct vs. contralateral samples (left columns) the lanes labelled with number 1–5 are contralateral samples and lanes labelled number 6–10 are their respective peri-infarct samples. The lanes marked with X were test samples used for the accuracy of the experiment and were not included for statistical analysis; therefore, they were omitted in final figures. In all contralateral versus ischemic core samples (right columns), the lanes labelled number 1–7 are contralateral samples and the lanes labelled number 8–14 are their respective ischemic core samples. Arrows show the band for the protein of interest according to molecular weight. The other bands are nonspecific which could be due to the many different cell types in brain tissue. Molecular weight marker was loaded in all experiments. M molecular marker. [file 12868_2022_696_MOESM2_ESM.pdf]

Contralateral (lane label 1-5) Peri-infarct (lane label 6-10)

3A

p-ERK1/2 Correspond to figure 3A in the manuscript

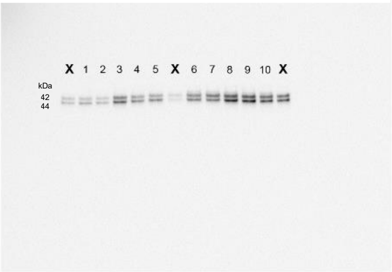

t-ERK1/2 Correspond to figure 3A in the manuscript

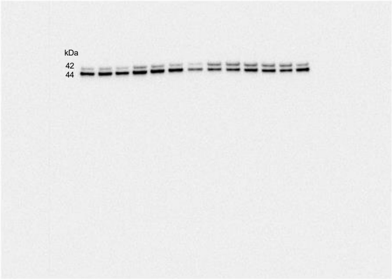

β-actin Correspond to figure 3A in the manuscript

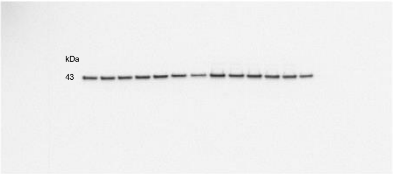

Contralateral (lane 1-7) Ischemic core (lane 8-14)

p-ERK1/2 Correspond to figure 3A in the manuscript

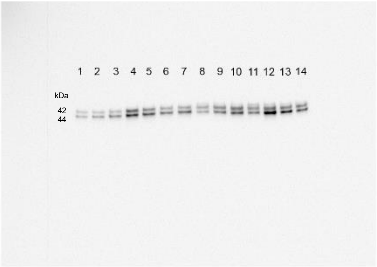

t-ERK1/2 Correspond to figure 3A in the manuscript

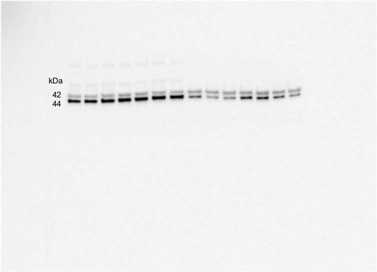

β-actin Correspond to figure 3A in the manuscript

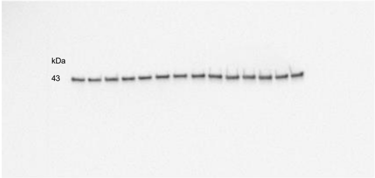

Contralateral (lane label 1-5) Peri-infarct (lane label 6-10)

3C

p-AKT Correspond to figure 3C in the manuscript

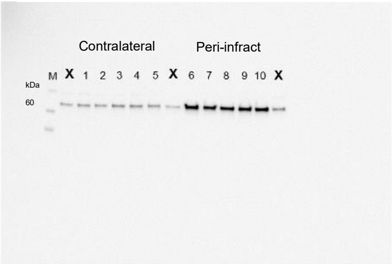

AKT Correspond to figure 3C in the manuscript

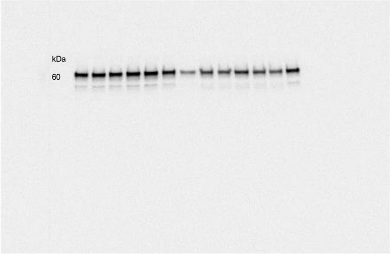

β-actin Correspond to figure 3C in the manuscript

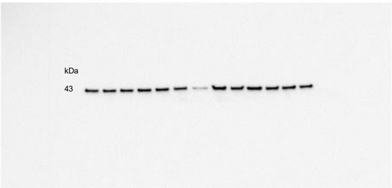

Contralateral (lane 1-7) Ischemic core (lane 8-14)

p-AKT Correspond to figure 3C in the

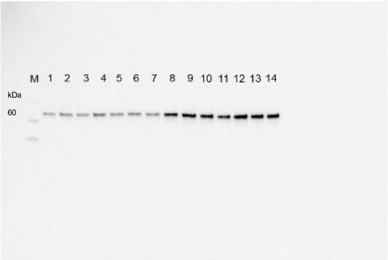

AKT Correspond to figure 3C in the manuscript

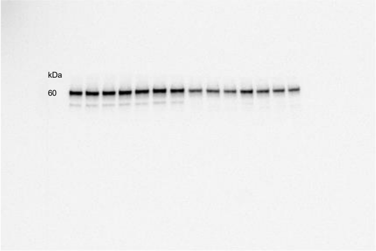

β-actin Correspond to figure 3C in the manuscript

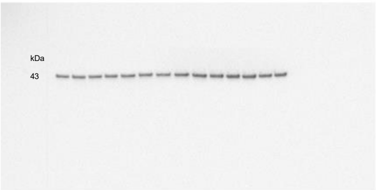

Figure Supporting Information S2

Raw images for Western blot membranes corresponding to figure 3A and 3C in the manuscript.

3A: phosphorylated ERK1/2, total ERK1/2 and β-actin corresponding to figure 3A in the manuscript.

3C: phosphorylated Akt, total Akt, and β-actin corresponding to figure 3A in the manuscript.

In all peri-infarct vs. contralateral samples (left columns) the lanes labelled with number 1 to 5 are contralateral samples and lanes labelled number 6 to 10 are their respective peri-infarct samples. The lanes marked with X were test samples used for the accuracy of the experiment and were not included for statistical analysis; therefore, they were omitted in final figures.

In all contralateral versus ischemic core samples (right columns), the lanes labelled number 1 to 7 are contralateral samples and the lanes labelled number 8 to 14 are their respective ischemic core samples. Arrows show the band for the protein of interest according to molecular weight. The other bands are nonspecific which could be due to the many different cell types in brain tissue. Molecular weight marker was loaded in all experiments. M; molecular marker.
