# Supplementary material for: Repair-related molecular changes during recovery phase of ischemic stroke in female rats
Source: BMC Neurosci. 2022 Apr 12;23:23. doi: 10.1186/s12868-022-00696-x (PMC9004052; doi:10.1186/s12868-022-00696-x)
Supplement: Supplementary file 3 — Additional file 3: Figure S2. Raw images for Western blot membranes corresponding to Fig. 4A in the manuscript. 3A: Nestin, NeuN and β-actin corresponding to Fig. 4C in the manuscript. In all contralateral samples versus peri-infarct (left columns) the lanes labelled with number 1–5 are contralateral samples and lanes labelled number 6–10 are their respective peri-infarct samples. The lanes marked with X were test samples used for the accuracy of the experiment and were not included for statistical analysis; therefore, they were omitted in final figures. In all contralateral versus ischemic core samples (right columns), the lanes labelled number 1–7 are contralateral samples and the lanes labelled number 8–14 are their respective ischemic core samples. Arrows show the band for the protein of interest according to molecular weight. The other bands are nonspecific which could be due to the many different cell types in brain tissue. Molecular weight marker was loaded in all experiments. M molecular marker. [file 12868_2022_696_MOESM3_ESM.pdf]

# Corresponding to Figure 4A

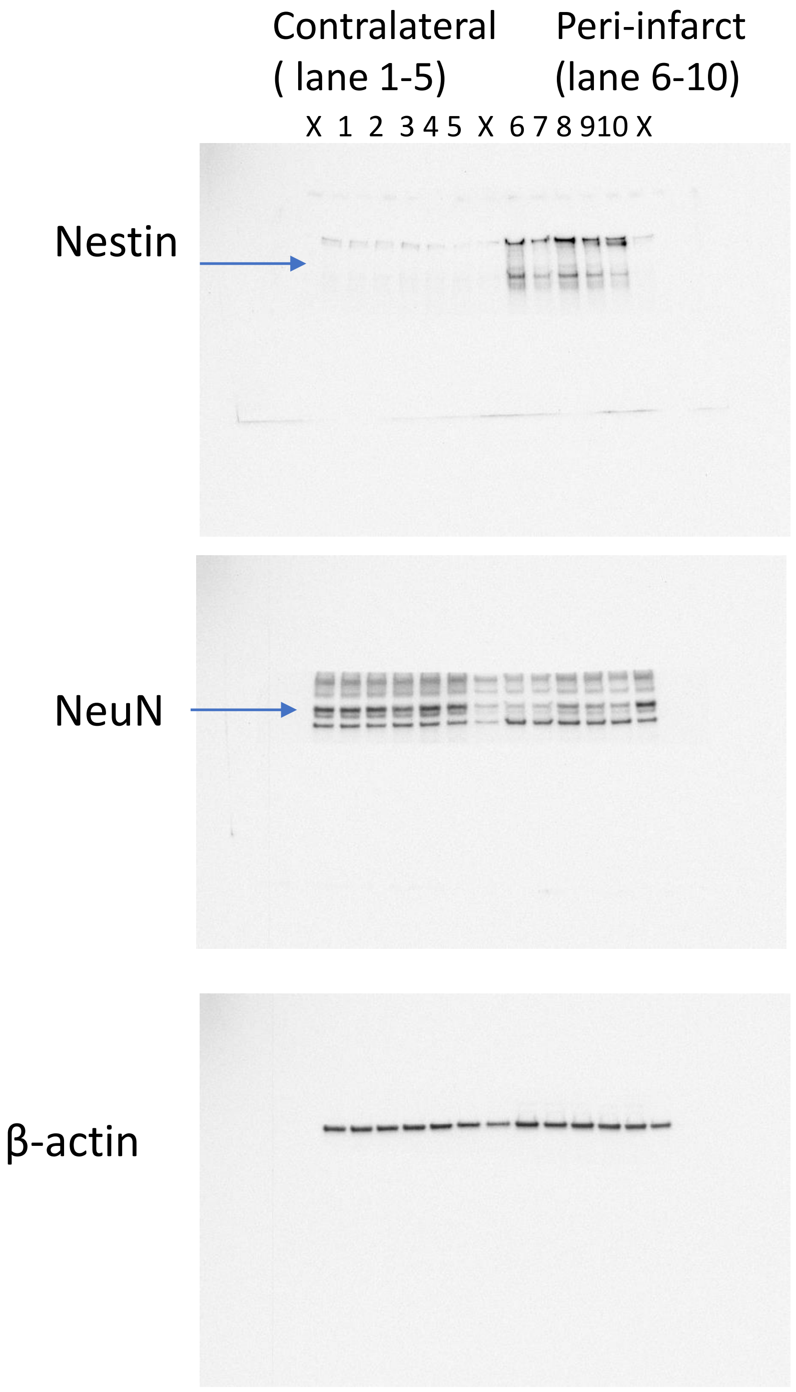

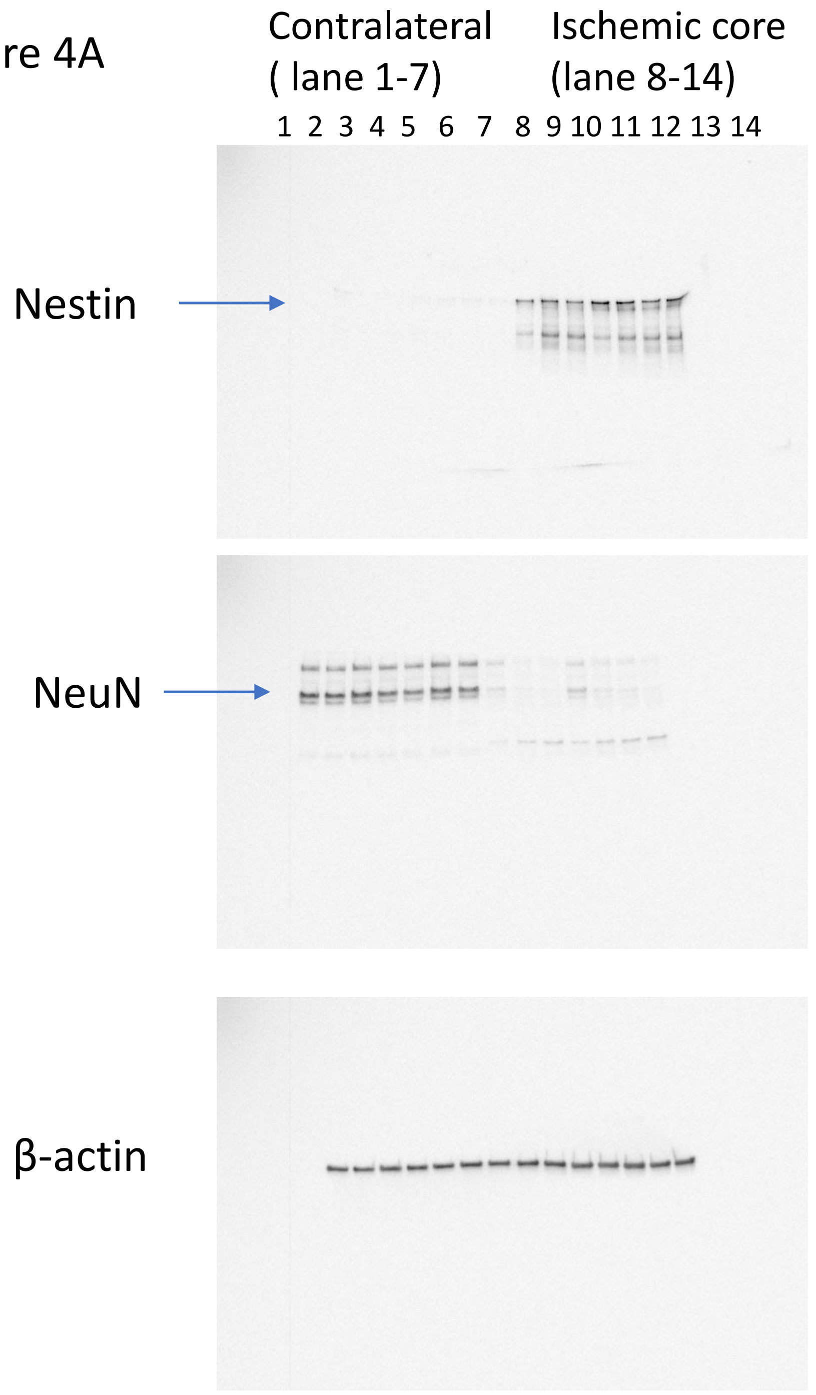

Figure Supporting Information S3

Raw images for Western blot membranes corresponding to figure 4A in the manuscript.

4A: Nestin, NeuN and β-actin corresponding to figure 4C in the manuscript.

In all contralateral samples versus peri-infarct (left columns) the lanes labelled with number 1 to 5 are contralateral samples and lanes labelled number 6 to 10 are their respective peri-infarct samples. The lanes marked with X were test samples used for the accuracy of the experiment and were not included for statistical analysis; therefore, they were omitted in final figures.

In all contralateral versus ischemic core samples (right columns), the lanes labelled number 1 to 7 are contralateral samples and the lanes labelled number 8 to 14 are their respective ischemic core samples. Arrows show the band for the protein of interest according to molecular weight. The other bands are nonspecific which could be due to the many different cell types in brain tissue. Molecular weight marker was loaded in all experiments. M; molecular marker.
